# Impact of an Engineered Copper-Titanium Dioxide Nanocomposite and Parent Substrates on the Bacteria Viability, Antioxidant Enzymes and Fatty Acid Profiling

**DOI:** 10.3390/ijms21239089

**Published:** 2020-11-29

**Authors:** Oliwia Metryka, Daniel Wasilkowski, Anna Nowak, Małgorzata Adamczyk-Habrajska, Agnieszka Mrozik

**Affiliations:** 1 Doctoral School, University of Silesia, Bankowa 14, 40-032 Katowice, Poland; 2 Institute of Biology, Biotechnology and Environmental Protection, Faculty of Natural Sciences, University of Silesia, Jagiellońska 28, 40-032 Katowice, Poland; agnieszka.mrozik@us.edu.pl; 3 Institute of Nuclear Physics, Polish Academy of Sciences, PL-31342 Kraków, Poland; anna.nowak@ifj.edu.pl; 4 Institute of Materials Engineering, Faculty of Science and Technology, University of Silesia, Żytnia 12, 41-200 Sosnowiec, Poland; malgorzata.adamczyk-habrajska@us.edu.pl

**Keywords:** antimicrobial activity, antioxidant enzymes, bacteria, Cu/TiO_2_ nanocomposite, Cu and TiO_2_ nanoparticles, FAME profiling, MARA test

## Abstract

Due to the systematic increase in the production of nanomaterials (NMs) and their applications in many areas of life, issues associated with their toxicity are inevitable. In particular, the performance of heterogeneous NMs, such as nanocomposites (NCs), is unpredictable as they may inherit the properties of their individual components. Therefore, the purpose of this work was to assess the biological activity of newly synthesized Cu/TiO_2_-NC and the parent nanoparticle substrates Cu-NPs and TiO_2_-NPs on the bacterial viability, antioxidant potential and fatty acid composition of the reference *Escherichia coli* and *Bacillus subtilis* strains. Based on the toxicological parameters, it was found that *B. subtilis* was more sensitive to NMs than *E. coli.* Furthermore, Cu/TiO_2_-NC and Cu-NPs had an opposite effect on both strains, while TiO_2_-NPs had a comparable mode of action. Simultaneously, the tested strains exhibited varied responses of the antioxidant enzymes after exposure to the NMs, with Cu-NPs having the strongest impact on their activity. The most considerable alternations in the fatty acid profiles were found after the bacteria were exposed to Cu/TiO_2_-NC and Cu-NPs. Microscopic images indicated distinct interactions of the NMs with the bacterial outer layers, especially in regard to *B. subtilis*. Cu/TiO_2_-NC generally proved to have less distinctive antimicrobial properties on *B. subtilis* than *E. coli* compared to its parent components. Presumably, the biocidal effects of the tested NMs can be attributed to the induction of oxidative stress, the release of metal ions and specific electrochemical interactions with the bacterial cells.

## 1. Introduction

Nanotechnology offers new interdisciplinary solutions and unlimited worldwide development opportunities via the implementation of different nanoscale structures in various spheres of life. Nanoparticles (NPs) and nanomaterials (NMs), especially their inorganic counterparts, can have a great contribution in global advancements due to their unique physical, chemical and biological properties. Although nanostructures are associated with modern science, they were used in ancient Egypt, India, Greece and Rome for decorative purposes and to craft iridescent glassware. For example, in the 3rd century BC, Egyptian blue, the oldest known artificial pigment in human history, which consists of a mixture of nanosized CaCuSi_4_O_10_ and SiO_2_, was used in wall painting, tombs, furnishings and statues [1]. Nowadays, engineered NPs and nanostructured materials based on Cu and Ti transition metals have been used in the production of everyday products (cosmetics, medicine, clothes, paints) as well as in various industrial technologies (electronics and plastics production, vehicle manufacture, food production) [1,2]. A global industry analysis report for the forecast period 2018−2023 created by Report Consultant [3] showed that the global market for Cu-NPs in 2017 was 1447.79 million USD and is estimated to reach 2280.57 million USD by 2023. According to the Transparency Market Research report for the global industry of Cu-NPs market for 2017−2025 [4], the majority of Cu structures are used in the production of antimicrobials, electronics, plastics, textiles, coatings, inks, catalysts for chemical reactions or conductive coatings. By comparison, the global market for TiO_2_-NPs prepared by the Allied Market Research for 2014−2022 [5] was 3.4 billion USD in 2014 and is estimated to reach 16.682 billion USD by 2022. The largest market for these TiO_2_-NPs in consumer products is in the production of paints, coatings, pigments, cosmetics, plastics and textile fibres, with Asia Pacific being the leader market. The greatest share for Cu-NPs is in North America, while similar to TiO_2_-NPs, the greatest expansion is estimated for the Asia Pacific market. 

Recent research has focused on creating alternative and enhanced forms of nanocomposites (NCs) composed of either organic or inorganic counterparts or both, e.g., Au/TiO_2_, Cu/SiO_2_, CuO/TiO_2_ and Pd/Fe_3_O_4_ [6,7,8,9]. According to the Markets and Markets Ltd. Report, the global NCs market is expected to increase from 4.1 billion USD in 2019 to as much as 8.5 billion USD by 2024 [10]. Combining one or more inorganic components improves the characteristics and specific nano-functions of NCs by maintaining selected properties from the parent materials [8]. For example, apart from its effective antimicrobial activity, Cu/SiO_2_-NC is also characterized by the controlled release of metal ions from the nanostructure [9]. Amongst the different types of NCs, Cu/TiO_2_ has gained recognition in the scientific world due to its photolytic activity and enhanced antimicrobial properties [11]. TiO_2_-NPs are commonly used as a base for the adsorption of Cu-NPs onto their surface, which acts as an oxide support and stabilizer of Cu ions [11,12]. For example, doping TiO_2_ nanotubes with Cu-NPs significantly increased their photocatalytic activity and light absorption [13]. Additionally, Thongpool et al. [14] reported greater photolytic activity of Cu-N that had been co-doped with TiO_2_-NPs compared to pure TiO_2_-NPs. Moreover, the results obtained by Chen at al. [11] confirmed the synergistic antibacterial activity of Cu/TiO_2_-NC towards *E. coli* cells, which was much higher than their constituents. 

The growing abundance and dispersion of engineered NMs in ecological matrices as well as their high biological reactivity attributed to their specific nano effects, poses a concern for creating new target sites and possible hazards for the action of these materials. NMs at systematically increasing concentrations in the environment could pose a threat to populations of microorganisms, which are not the primary target of their activity. Therefore, it seems to be necessary to undertake advanced quantitative and qualitative studies in order to expand our knowledge about the nanotoxicological effects of NMs on the functioning of micro-organisms [2,15]. The direct and indirect negative impacts of inorganic NPs and NMs on microbial cells include: disrupting cellular integrity, causing cytoplasmic leakage, damage to the intracellular structures (ribosomes, nucleic acids, proteins), disrupting the respiratory cell functions (electron transport chain, protein depletion, ATP production) and causing cell necrosis [16,17]. However, the primary mode of action of metallic nanostructures is the generation of highly reactive oxygen species (ROS) such as superoxidase anion (O_2_^•^^−^), hydrogen peroxide (H_2_O_2_) and hydroxyl radical (^•^OH). The ROS that are generated induce oxidative stress in cells via catalytic oxidation, which causes lipid peroxidation and the loss of cell membrane integrity, the oxidation of proteins, and damage to the cell enzymatic system and nucleic acid molecules. The antioxidant protein catalysts of cells such as catalase, peroxidase and superoxide dismutase are one of the primary cell defence systems against ROS. Therefore, an imbalance between ROS synthesis and the antioxidant defence system, including free radical scavengers such as glutathione, is an important determinant of the toxicity of NPs and NMs [16,17]. 

Despite many attempts that have been made to assess and define the hazards associated with the risk of introducing NMs into the environment and their toxicity against microorganisms, these issues are still inadequately understood. The results of many case studies have shown contradictory results, thereby complicating nanotoxicological studies and making it difficult to understand the mechanisms of the action of nanostructures in cells [1,18]. Although oxidative stress in microbial cells caused by exposure to different NPs and NMs has been extensively studied, their effect on the activity of the antioxidant enzymes is still very limited. In studies of antioxidant enzymes, catalase activity is most often measured by authors [19,20,21,22,23,24], whereas there are very few studies concerning the effect of nanostructures on bacterial peroxidase activity [20,24,25]. It is also very important to stress that there is a large knowledge gap in understanding the impact of NPs and NMs on the cellular fatty acid profiles as well as the interaction of these structures with the cell surface. Given this, in order to analyse the effects of the newly synthesized Cu/TiO_2_-NC and its parent substrates Cu-NPs and TiO_2_-NPs on selected bacteria cells, the specific goals of this research were: (1) to determine the size and shape of the synthesized NMs, (2), to evaluate and compare their antimicrobial activity towards two reference *Escherichia coli* and *Bacillus subtilis* strains, (3) to measure the activity of the bacterial antioxidant enzymes, (4) to identify any changes in the fatty acid composition and (5) to analyse the distribution of NMs on the bacterial cell surface. Additionally, in order to study the toxicity of Cu/TiO_2_-NC, Cu-NPs and TiO_2_-NPs against 11 other taxonomically diverse microbial strains, the microbial assay for risk assessment (MARA) was also performed. 

## 2. Results

### 2.1. Size and Shape of the Synthesized Cu/TiO_2_-NC and Cu-NPs 

**Figure 1:**
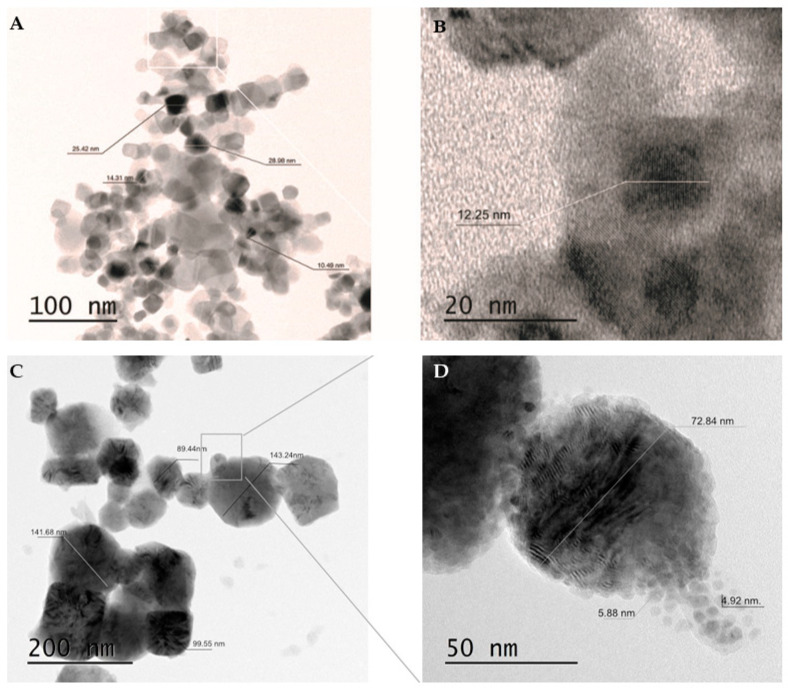
TEM micrographs of Cu/TiO_2_-NC using a two-scale bar: 100 nm (**A**) and 20 nm (**B**) and agglomerated Cu-NPs using a two-scale bar: 200 nm (**C**) and 50 nm (**D**).

The morphology and size of the newly synthesized Cu/TiO_2_-NC and parent Cu-NPs were characterized using a transmission electron microscope (TEM) (Figure 1). The sizes of Cu/TiO_2_-NC ranged from 12.35 ± 1.91 nm to 25.16 ± 0.37 nm. They had an irregular shape and were trapped in the TiO_2_ matrix (Figure 1A). The sizes of the synthesized Cu-NPs were in the range of 5.40 ± 0.68 nm to 110.22 ± 27.71 nm. Moreover, they had a tendency to agglomerate (Figure 1B). By comparison, the commercially available TiO_2_-NPs were characterized by a size of 20 nm and had diverse prism shapes (CAS No. 13463-67-7). 

### 2.2. Antibacterial Activity of Cu/TiO_2_-NC, Cu-NPs and TiO_2_-NPs

The antibacterial activity of all of the NMs was examined against two reference bacterial strains *Escherichia coli* ATCC 25922 and *Bacillus subtilis* ATCC 6633, which were purchased from the American Type Culture Collection and were widely used in toxicological studies. By applying model strains, standardization of the research and collective comparison of the results became possible. A traditional microbiological test based on the culture of bacteria in the presence of NMs was applied to determine the minimum bactericidal concentration (MBC), the minimum inhibitory concentration (MIC) and the half maximal inhibitory concentration (IC_50_) (Table 1). These parameters can be used as benchmarks for future toxicological studies by providing quality control and validating the obtained results. 

Generally, both strains proved to be sensitive to the presence of all of the tested NMs, however, specific NMs generated different responses from the bacterial cells. The obtained results clearly indicate that *E. coli* was more resistant to the stress caused by the tested NPs than *B. subtilis*, however, an opposite effect was observed for Cu/TiO_2_-NC. The antibacterial effect of NMs on *E. coli* cells was quite similar, with average MIC and MBC values that ranged from 500 to 600 mg L^−1^. Interestingly, Cu-NPs had a 5-fold lower lethal effect on the *E. coli* cells than TiO_2_-NPs (IC_50_ = 102.16 mg L^−1^). Based on the obtained IC_50_ values, the bactericidal effect of the tested NMs on the *E. coli* cells can be ordered as follows: Cu-NPs < TiO_2_-NPs < Cu/TiO_2_-NC. By comparison, *B. subtilis* exposed to NMs showed the opposite effect, with the greatest sensitivity to Cu-NPs. The calculated IC_50_ values of the tested NMs for *B. subtilis* increased in the following order: Cu/TiO_2_-NC < TiO_2_-NPs < Cu-NPs. The obtained results clearly indicated that the *E. coli* strain was more resistant to the stress caused by the tested NMs than *B. subtilis*. 

### 2.3. Impact of Cu/TiO_2_-NC, Cu-NPs and TiO_2_-NPs on the Activity of Catalase, Superoxide Dismutase and Peroxidase 

**Figure 2:**
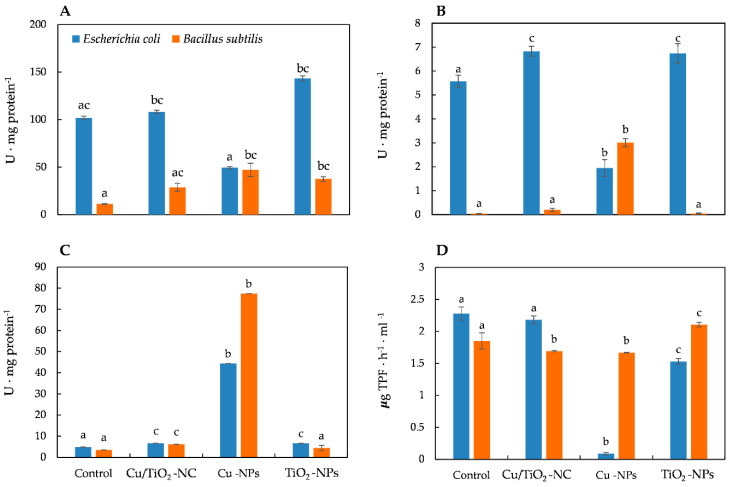
The activities of CAT (**A**), PER (**B**), SOD (**C**) and DEH (**D**) in *E. coli* and *B. subtilis* after treatment with NMs and in the control cells. Different letters indicate statistically significant differences (*p* < 0.05) among means between control and NMs treatment.

To study and compare the impact of Cu/TiO_2_-NC, Cu-NPs and TiO_2_-NPs on the generation of oxidative stress in *E. coli* and *B. subtilis*, the activities of the triad enzymes of the antioxidant defence system including catalase (CAT), peroxidase (PER) and superoxide dismutase (SOD) as well as the overall dehydrogenase activity (DEH) were measured. The statistical analyses showed that all of the NMs significantly affected the activity of all of the enzymes (ANOVA, Tukey’s test) in both bacterial strains. Catalase, which catalyses the breakdown of H_2_O_2_ to O_2_ and H_2_O and thereby protects cells from the oxidative damage caused by ROS, is a common enzyme in all living organisms. Interestingly, the addition of Cu/TiO_2_-NC to the *E. coli* culture resulted in a slight increase in the activity of CAT (108.09 U ⋅ mg protein^−1^) compared to its activity in the control cells (101.73 U ⋅ mg protein^−1^). In turn, the highest increase in the CAT activity (about 1.4-fold) was observed after a 24-hour exposure of the cells to TiO_2_-NPs. Conversely, in the presence of Cu-NPs, there was a 2-fold decrease in the CAT activity in *E. coli* (Figure 2A). By comparison, *B. subtilis* exhibited the highest (3- to 4-fold) increase in the CAT activity after treatment with Cu-NPs and TiO_2_-NPs (Figure 2A). The obtained results indicate that CAT had a greater sensitivity to the tested NMs in *B. subtilis* than in *E. coli*. 

In a parallel experiment, the activity of PER, which catalyses the reduction of H_2_O_2_ to water and oxygen, was calculated. Exposure of the *E. coli* cells to Cu/TiO_2_-NC and TiO_2_-NPs resulted in a noticeable increase in the PER activity, while the addition of Cu-NPs resulted in its significant decrease (about 3-fold) compared to the PER activity in the control cells (Figure 2B). Similarly, the addition of the tested NMs to the *B. subtilis* culture had a stimulating effect on the activity of PER, which resulted in the highest increase (about 70-fold) after treatment with Cu-NPs (Figure 2B). Interestingly, in *E. coli*, PER was more sensitive to the tested NMs than PER in *B. subtilis* cells, however, the functioning of PER from both strains proved to be most affected by the presence of Cu-NPs.

Measurements of SOD activity also showed involvement of this enzyme in the catalysis of dismutation of O_2_^•−^ to O_2_ and H_2_O_2_, based on the response of *E. coli* and *B. subtilis* to the tested NMs. The calculated SOD activities gave rise to the conclusion that SOD in *E. coli* was less active than in *B. subtilis* after exposure to NMs. The SOD activity in *E. coli* was slightly higher than in the control cells after treatment with Cu/TiO_2_-NC and TiO_2_-NPs; however, it drastically increased (about 9-fold) in the cells exposed to Cu-NPs (Figure 2C). By comparison, *B. subtilis* exhibited a 1.25-, 1.78- and 22-fold higher SOD activity than in the control cells after Cu/TiO_2_-NC, TiO_2_-NPs and Cu-NPs were added to a culture, respectively (Figure 2C).

It is worth emphasising that almost all of the tested NMs decreased the DEH activity in both strains. The function of DEH is to catalyse the oxidation-reduction reactions required for the respiration of organic compounds. The dehydrogenases in *E. coli* were most sensitive to Cu-NPs, less sensitive to TiO_2_-NPs and least sensitive to Cu/TiO_2_-NC, achieving about 4%, 67% and 96% of their activity as in the control cells, respectively (Figure 2D). By comparison, the DEH activity in *B. subtilis* was most affected by Cu-NPs and Cu/TiO_2_-NC; however, their activity was higher than in *E. coli*, and reached about 90% of their activity as in the nontreated cells. Simultaneously, the DEH activity in the presence of TiO_2_-NPs increased by 14% compared to their activity in the control cells (Figure 2D). To summarize, in *E. coli*, DEH were affected more by the tested NMs than DEH in *B. subtilis*.

### 2.4. Effects of Cu/TiO_2_-NC, Cu-NPs and TiO_2_-NPs on the Bacterial Fatty acid Composition

**Figure 3:**
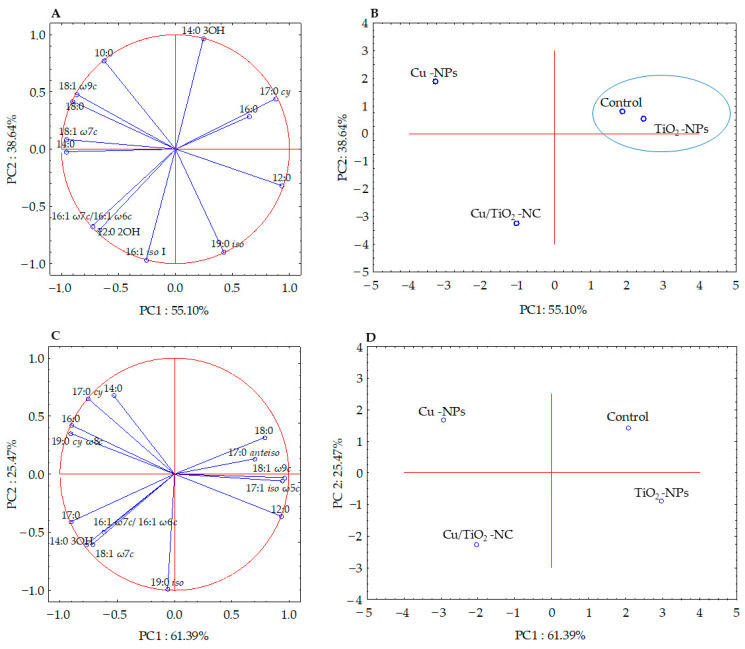
Projection of the individual fatty acids along PC1 and PC2 in the FAME profiles of *E. coli* (**A**,**B**) and *B. subtilis* (**C**,**D**) treated with NMs and in the control cells. The samples with similar PC1 and PC2 values are included in a cluster.

The fatty acid methyl esters (FAMEs) data obtained for each bacterial strain were subjected to the selection of a subset of significant fatty acids (ANOVA, *p* > 0.05) in order to illustrate the differences and similarities between the tested samples. Six of the thirteen high-loading FAMEs of *E. coli* were analysed using PCA, while the statistically not significant (*p* > 0.05) fatty acids (10:0, 12:0, 12:0 2OH, 14:0, 16:0, 19:0 *iso* and 18:1 ω7*c*) were omitted. The factor analysis provided two factors that explained 93.74% of the variance (Figure 3A). Fatty acids that correlated most with PC1 were the 12:0, 14:0, 17:0 *cy,* 18:0, 18:1 ω7*c* and 18:1 ω9*c*, whereas, the 14:0 3OH, 19:0 *iso* and 16:1 *iso* I fatty acids correlated with PC2. The projections of the FAME profiles distinguished the samples along the PC1 and PC2 axes. The samples treated with Cu/TiO_2_-NC and Cu-NPs formed separate clusters and were positively correlated with 16:1 ω7*c*/16:1 ω6*c*, 10:0, and 12:0 2OH, respectively. In turn, the sample exposed to TiO_2_-NPs and the control sample were grouped together along PC1 and PC2, and were positively correlated with 16:0 and 17:0 *cy* (Figure 3B).

Simultaneously, the analysis of the FAME profiles of *B. subtilis* clearly indicated that there was no significant correlation between the fatty acids isolated from the control cells and the cells treated with NMs. The PCA projection of eleven fatty acids showed 86.86% of the variance (Figure 3C). Along PC1, all of the samples were grouped separately, while along PC2, the control sample and the sample treated with Cu-NPs as well as the samples exposed to Cu/TiO_2_-NC and TiO_2_-NPs were grouped together (Figure 3D). The biplot graphic revealed a strong positive correlation of 18:1 ω7*c*, 17:0 *cy*, 12:0 and 18:0 with Cu/TiO_2_-NC_,_ Cu-NPs, TiO_2_-NPs and the control sample, respectively (Figure 3C-D). 

**Figure 4:**
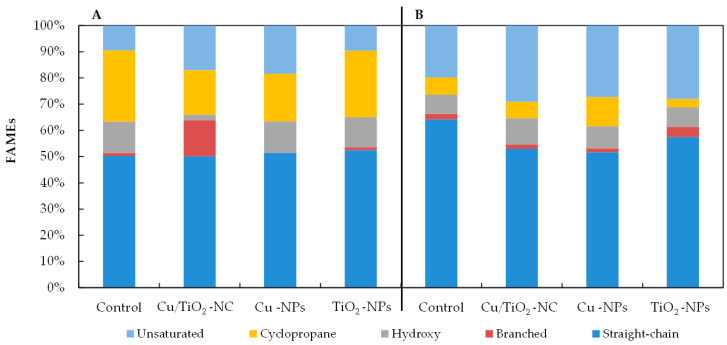
The percentages of straight-chain, branched, hydroxyl, cyclopropane and unsaturated fatty acids in the FAME profiles of *E. coli* (**A**) and *B. subtilis* (**B**) treated with Cu/TiO_2_-NC, Cu-NPs, TiO_2_-NPs and in the control cells.

As Figure 4 illustrates, all of the NMs had a significant impact on the percentages of the individual groups of fatty acids in the tested bacteria. The observed changes in the fatty acid content depended on the type of nanomaterial and the species of bacteria. For example, there was a relatively high increase of 7.55% and 8.88% in the unsaturated fatty acid abundance in the FAME profiles of the *E. coli* exposed to Cu/TiO_2_-NC and Cu-NPs, respectively, compared to the control cells (Figure 4A). Moreover, when these bacteria were treated with Cu-NPs and Cu/TiO_2_-NC, it resulted in a considerable decrease of 8.96% and 10.13% in the abundance of the cyclopropane fatty acids, respectively, compared to the nontreated cells. Interestingly, in the presence of Cu/TiO_2_-NC, the bacteria changed the content of the branched fatty acids by 12.6% compared to the control cells; however, after the Cu-NP treatment, these fatty acids were not detected. By comparison, in the FAME profiles of *B. subtilis*, the greatest decrease in the fatty acid proportions of 11.24% and 12.55% were found in the straight-chain fatty acids in the cells treated with Cu/TiO_2_-NC and Cu-NPs, respectively (Figure 4B). These decreases were accompanied by increases in the unsaturated fatty acid content. Similar to *E. coli, B. subtilis* increased the content of cyclic fatty acids by 1.7-fold compared to the nontreated cells after Cu-NP treatment; however, in the presence of TiO_2_-NPs, there was a 2-fold lower content of these acids. The analyses showed that the NMs that had the greatest impact on the fatty acid profiles of the tested bacteria were Cu/TiO_2_-NC and Cu-NPs.

### 2.5. SEM-EDS Images 

**Figure 5:**
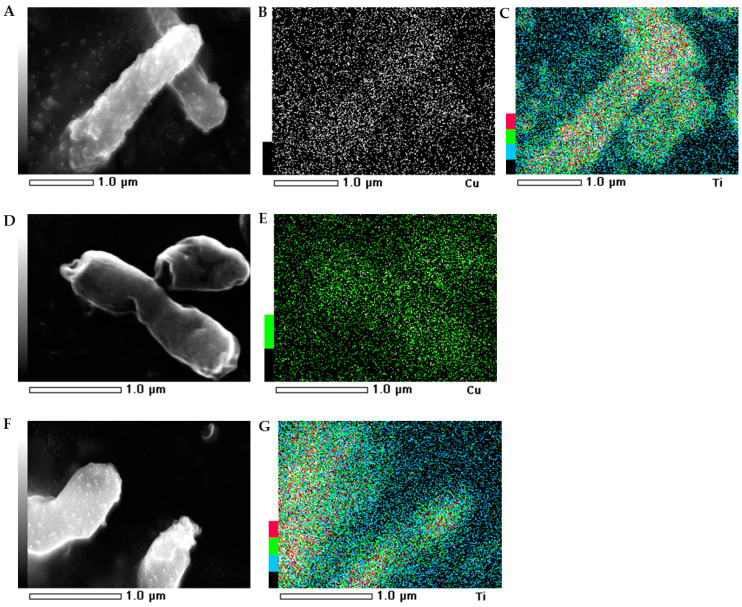
SEM-EDS photographs of the *E. coli* treated with Cu/TiO_2_ -NC (**A**); Cu-NPs (**D**) and TiO_2_-NPs (**F**). EDS micrographs of selected elements: Cu (**B**,**E**) and Ti (**C**,**G**).

**Figure 6:**
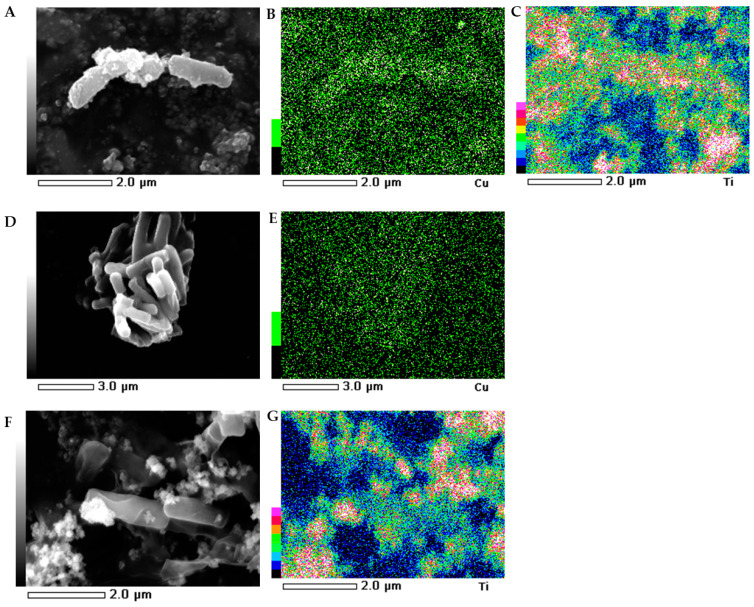
SEM-EDS photographs of the *B. subtilis* treated with Cu/TiO_2_ -NC (**A**); Cu-NPs (**D**) and TiO_2_-NPs (**F**). EDS micrographs of selected elements: Cu (**B**,**E**) and Ti (**C**,**G**).

The morphology of the bacterial strains treated with NMs were visualized individually using scanning electron microscopy (SEM). Additionally, the mapping of the cell surface at the microstructural level was analysed using SEM with energy dispersive X-ray spectrometry (EDS). The SEM images indicated that the morphology of the control *E. coli* and *B. subtilis* cells was typical and intact/integrated (Figure 5 and Figure 6). Simultaneously, the SEM-EDS analyses showed that the tested NMs had a strong affinity to the cell surfaces of *E. coli* and *B. subtilis*. Interestingly, although Cu/TiO_2_ -NC and TiO_2_ -NPs were uniformly dispersed around the entire surface of the *E. coli* cells (Figure 5A−C,F−G), they exhibited a high affinity to the peripheral cell site of the *B. subtilis* cells where they formed agglomerates (Figure 6A−C,F−G). Moreover, the SEM micrographs of the *B. subtilis* treated with Cu/TiO_2_-NC indicated an impaired ability of the bacteria to form biofilm compared to the control image due to the dominance of single cells and the observed small clusters of cells (Figure 6A). Conversely, in the presence of Cu-NPs and TiO_2_-NPs, the bacterial population retained the ability to form biofilm (Figure 6D,F).

### 2.6. Microbial Assay for Risk Assessment (MARA) 

To assess the antimicrobial activity of the tested NMs against 11 other microbial species: *Microbacterium* spp., *Brevundimonas diminuta*, *Citrobacter freundii*, *Comamonas testosterone*, *Enterococcus casseliflavus*, *Delftia acidovorans*, *Kurthia gibsonii*, *Staphylococcus warneri*, *Pseudomonas aurantiaca*, *Serratia rubidaea* and the yeast *Pichia anomala*, the microbial assay for risk assessment (MARA), which is based on a unique and multidimensional assay with a microbial toxic concentration index (MTC) was used. The obtained results clearly indicated that there was a different sensitivity of the various phylogenetic strains only to Cu-NPs (Table 2). Considering MTC_av._ = 559 mg L^−1^, it was confirmed that Cu-NPs had the greatest bactericidal effect on the Gram-positive strains (MTC < MTC_av._). Interestingly, both Cu/TiO_2_-NC and TiO_2_-NPs showed no toxicological properties on the tested microorganisms (MTC > 1000 mg L^−1^), with the exception of Cu/TiO_2_-NC, which induced a low bactericidal effect on *Kurthia gibsonii*. It is worth mentioning that none of the NMs showed an antifungal activity against *Pichia anomala*. 

## 3. Discussion

The risks of the commercialisation of materials with nanoscale components are the subject of ongoing discussion in the scientific community. The question is whether the great opportunities of using engineered NMs outweigh the unknown actions of these structures on nontarget microorganisms. The toxicity of NMs requires an assessment of their basic physicochemical parameters, including their size, chemical composition, shape and surface charge as well as their ability to form aggregates and agglomerates that are responsible for their activity. Therefore, in this study, a TEM analysis was performed in order to determine the morphology and size of the newly synthesized Cu-NPs and Cu/TiO_2_-NC. The obtained micrographs indicated that both NMs had a tendency to form irregular agglomerates with individual particle sizes ranging from 5.40 ± 0.68 nm to 110.22 ± 27.71 nm. The phenomena of aggregation and agglomeration may contribute to lowering the biocidal properties of NMs. Considering this, each time before the NMs were used, the stock solutions were subjected to ultrasound sonication in order to minimize these phenomena and obtain NMs that had a high biological activity.

The field of nanotoxicology is constantly evolving as many newly engineered NMs are being produced every day. Before their practical application, their possible toxicity should be thoroughly investigated because each NM is characterized by its own distinct physicochemical properties and mode of action. Currently, one of the most widely used methods for testing the adverse effects of these structures on bacteria is the use of reference bacterial strains. Here, the conducted toxicological study showed a differentiated impact of Cu/TiO_2_-NC, Cu-NPs and TiO_2_-NPs on the viability of *E. coli* and *B. subtilis*, which possibly resulted from the different interactions of the NMs with the bacterial outer layers of the Gram-negative and Gram-positive bacteria. Although the newly synthesized Cu/TiO_2_-NC proved to be more toxic to *E. coli* than to *B. subtilis*, its TiO_2_ component had a similar antibacterial effect against both strains. On the other hand, Cu-NPs proved to be more toxic against *B. subtilis*. This may be associated with the greater stability of Cu/TiO_2_-NC and the slower release of harmful metal ions from its surface compared to the pure parent Cu-NPs and TiO_2_-NPs [9]. In a similar study, Ruparelia et al. [26] confirmed a greater sensitivity of *B. subtilis* to Cu-NPs (20 and 40 mg L^−1^, respectively) than four *E. coli* strains (140−280 and 160−300 mg L^−1^, respectively) based on their MIC and MBC values. In this work, *B. subtilis* was also more susceptible to Cu-NPs compared to *E. coli*. Its greater sensitivity might be attributed to the increased affinity of the Cu ions to the amino acids and carboxyl groups on the surface of these bacteria [26,27]. Although an antibacterial effect of Cu-NPs on *E. coli* strain was also revealed by Kaweeteerawat et al. [28], the IC_50_ value (120 ± 14 mg L^−1^) was 4.2 times lower than the IC_50_ calculated for *E. coli* in this study. Although the biocidal activity of TiO_2_-NPs has been tested against many bacteria, the results also differ significantly from those that were obtained in this work. For example, in the study by Tong et al. [29], the antibacterial activity of TiO_2_-NPs against *E. coli* ATCC 25922 was found to be almost 20-fold lower than against the tested *E. coli*, while in the research of Suppi et al. [30] and Bonnet et al. [31], it was only 2-fold and 0.3-fold weaker, respectively. In the case of Cu/TiO_2_-NC, Chen et al. [11] reported a 5-fold lower biocidal activity against *E. coli* compared to the parent Cu-NPs, which was similar to our results. Herein, the opposite effect of Cu/TiO_2_-NC on *B. subtilis* might be attributed to its tendency to agglomerate and aggregate with the substances in a medium and the compounds secreted by bacterial cells. In addition, the ability of *B. subtilis* to form biofilm might provide supplementary protection against NC action.

The biocidal activity of NMs is usually attributed to the induction of oxidative stress and a disruption in the functioning of antioxidants. The presented research showed that Cu/TiO_2_-NC, Cu-NPs and TiO_2_-NPs had diverse effects on the activity of the tested enzymes. Generally, the addition of Cu/TiO_2_-NC, Cu-NPs and TiO_2_-NPs to the *E. coli* and *B. subtilis* cultures stimulated the activity of CAT, PER and SOD. Interestingly, the activity of CAT and PER in *E. coli* was significantly lower after exposure to Cu-NPs than those in *B. subtilis*. The greater antibacterial effect of Cu-NPs compared to Cu/TiO_2_-NC might be due to the release of Cu^2+^ active ions, which induce the formation of H_2_O_2_ [17,32]. It is probable that the lack of the observed effect of Cu/TiO_2_-NC on the activity of the tested enzymes in this study resulted from the phenomenon of homoagglomeration, which leads to the formation of larger structures that have lower antibacterial properties. In light of the obtained results, it can be concluded that the stimulation of the catalytic activity of CAT, PER and SOD in *E. coli* and *B. subtilis* could be the cell defence mechanism against the ROS generated by the tested NMs. A similar trend was also observed by Pal et al. [33] for *E. coli* exposed to Ag-NPs. The presented results are novel and scientifically valuable because of the very limited number of reports concerning the effect of NMs on the activity of bacterial CAT, PER and SOD. To date, the available papers are concerned only with the impact of Ag-NPs on the CAT, PER and SOD activity in *E. coli*, *B. cereus* and *Pseudomonas aeruginosa* [20,24]; Au-NPs on the CAT and SOD activity in *E. coli* [23]; SiC/Ag/cellulose nanocomposite on the CAT activity in *E. coli* and *B. subtilis* [21]; SiC nanofibers on the PER activity in *Pseudomonas putida* [25]; GO@CS/ZnO nanocomposite and Ag-NPs on the CAT activity in *Staphylococcus aureus* [19] and AgCl@SBA-15/IL on the CAT activity in *E. coli* [22]. 

The induction of oxidative stress and the generation of ROS by concentrations of NMs above the physiological state can lead to protein damage and lipid peroxidation, which consequently cause damage to the cell membrane, the respiratory chain and disturb the respiratory metabolism. A commonly used indicator for assessing the intensity of respiratory metabolism in living cells is to measure the activity of DEH. Due to the fact that DEH are a group of various enzymes, located in the cytoplasm and cytoplasmic membranes, they participate in the transfer of electrons to the redox transporters, thereby playing an important role in cell respiration. They can also be a useful biomarker for evaluating the physiological state of cells under oxidative stress, e.g., stress caused by NMs [34,35]. Although it was found that Cu/TiO_2_-NC slightly decreased the activity of DEH in *E. coli* and *B. subtilis*, the Cu-NPs decreased it more intensively with the exception of TiO_2_-NPs, which induced the activity of DEH in *B. subtilis*. In accordance with the calculated activities, DEH in *E. coli* proved to be more sensitive to NMs than DEH in *B. subtilis*. Greater sensitivity of DEH from Gram-negative *E. coli* may be attributed to the different structure of the outer layers compared to Gram-positive *B. subtilis*. Moreover, released metal ions from NMs could interact with enzyme thiol groups, consequently disrupting their functioning [35]. Similar to the sparingly studied activity of a triad of antioxidant enzymes in pure bacterial strains exposed to NMs, relatively little information is available on the DEH activity in such strains. The examples from the literature concern the lower activity of DEH in *P. aeruginosa* and *S. aureus* treated with Ag-NPs [35], a stimulation of the DEH activity in *E. coli* and *B. cereus* using SiC nanofibers as well as the inhibition of this enzyme in *B. cereus* using SiC/Ag/CE/T [21]. In another study, Borkowski et al. [36] reported that MgO-NPs strongly stimulated the DEH activity in *E. coli* and *B. cereus*, while SiO_2_-NPs only affected the *E. coli* enzymes. The provided examples clearly indicate the diverse impact of tested NMs on various bacterial strains.

Even though the stimulated activity of antioxidant defence system enzymes protects bacterial cells against the harmful effects of ROS, and hence, the antimicrobial activity of NMs, it cannot prevent or diminish their impact on other cellular components [37]. One of the outcomes of the negative impact of Cu/TiO_2_-NC, Cu-NPs and TiO_2_-NPs on the tested bacterial strains were changes in the composition and content of the distinct groups of fatty acids. In the presence of all NMs, both strains had an increased proportion of unsaturated fatty acids compared to the FAME profiles of the nontreated cells. Additionally, both *E. coli* and *B. subtilis* had a lower percentage of cyclopropane and saturated straight-chain fatty acids than the control cells. It is worth emphasising that Cu/TiO_2_-NC influenced the composition of the cellular fatty acids unexpectedly strongly despite the formation of large molecules. This could be explained by the strong electrostatic attraction between Cu/TiO_2_-NC and the negatively charged surface of the bacteria. In turn, the increased sensitivity of tested strains to Cu-NPs can be explained by an increase in the unsaturated fatty acid abundance in FAME profiles, which increased the fluidity and permeability of the cell membrane. By comparison, slight changes in the *E. coli* and *B. subtilis* FAME profiles after exposure to TiO_2_-NPs could have resulted from their affinity to form larger agglomerates and aggregates, thereby decreasing their binding affinity to the outer layers of bacterial cells. The observed changes in the fatty acid composition might be related to an impairment or damage to the cell membrane or ROS-induced lipid peroxidation, which can cause the leakage of the intracellular contents and ultimately the death of bacterial cells. Additionally, the interaction of Cu/TiO_2_-NC, Cu-NPs and TiO_2_-NPs with the outer layers and bacterial DEH can lead to cytoplasmic leakage and a disruption of the intracellular content [32,35]. Unfortunately, there is a very limited number of studies regarding the effect of similar NMs on the cellular fatty acid composition of single strains of bacteria, using the FAME technique. The only available information concerns the effect of different types of NMs, i.e., single-walled carbon nanotubes (SWCNT) and multiwalled carbon nanotubes (MWCNT), on the cellular fatty acid content of *E. coli*, *B. subtilis* and *S. aureus* [38]; TiO_2_-NPs on the fatty acid distribution in intestinal microorganisms [39]; and Ag-NPs, Cu-NPs, SiO_2_-NPs, Au-NPs, Cu-NPs, Pd-NPs and Si-NPs on the fatty acid alternations in soil microorganisms [40,41]. 

Microorganisms are characterized by a complex set of responses to stressful conditions including the presence of NMs. The adhesion of NMs to the cell surface can lead to the deformation of the cell wall and a disruption of the integrity of the cytoplasmic membrane due to the oxidation of the double bonds in the phospholipid fatty acids. Here, the micrographs confirmed the ability of the tested NMs to adhere to the surface of the *E. coli* and *B. subtilis* cells. It is worth noting that each of the NMs was characterized by a specific pattern of distribution on the surface of the bacteria, changing their morphology from smooth to slightly wrinkled (folded). The preserved and integrated structure of the cell wall can indicate that the effect of tested NMs on the bacteria was not only the result of the surface action but might also be dependent on their penetration into the cells. This suggestion is in agreement with Bao et al. [42], who observed a similar effect of Ag-NPs on *E. coli* cells. Based on the SEM analysis, it can also be concluded that Cu/TiO_2_-NC and TiO_2_-NPs spread over the cell surface of *E. coli* more evenly and were able to interact with the chemical groups that were present on its surface more than on the surface of *B. subtilis.* This was due to the fact that NMs were concentrated in the peripheral part of the *B. subtilis* cells. Interestingly, Cu/TiO_2_-NC reduced the ability of *B. subtilis* to form biofilm. By comparison, Kumar et al. [43] observed single TiO_2_-NPs clusters on the surface of *E. coli* cells using SEM analysis. Similarly, Leung et al. [44] visualized *E. coli* cell damage after exposure to TiO_2_-NPs and ZnO-NPs with apparent deformations and holes on the surface of the bacterial cells. Moreover, TiO_2_-NPs and ZnO-NPs formed clusters over the entire surface of the bacterial cells. By contrast, Li et al. [45] did not confirm the impact of catechin-modified Cu-NPs on the morphology of *E. coli* cells. 

A novelty of this work was the assessment of the toxicity of Cu/TiO_2_-NC, Cu-NPs and TiO_2_-NPs against 11 phylogenetically different microbial strains using the MARA test. Of all of the NMs, only the Cu-NPs proved to be highly toxic against the tested microorganisms. It is worth emphasising that despite its minor antibacterial activity, Cu/TiO_2_-NC proved to have a highly toxic effect on *K. gibsonii*, which is commonly present in various meats, milks and soils. However, none of the tested NMs displayed any antifungal properties. This partially verifies the potential use of Cu/TiO_2_-NC in future commercial products. Although the MARA assay is an easily accessible and uncomplicated method for assessing NM toxicity, there are currently only a limited number of studies that have used it [46,47].

## 4. Materials and Methods

### 4.1. Synthesis of Cu-NPs and Cu/TiO_2_-NC

The Cu-NPs that were required for the synthesis of Cu/TiO_2_-NC were synthesized in accordance with Nowak et al. [48]. A 10% portion of NaOH was added dropwise to an aqueous solution of copper (II) acetate (Avantor Performance Materials Poland S.A.), which had been selected as the donor of the Cu^2+^ ions. Next, l-ascorbic acid (Avantor Performance Materials Poland S.A.), which is a reducer of Cu^2+^ ions, was added to the prepared solution. The reaction was performed in controlled conditions (atmosphere: air, temperature ~80 °C). To synthesize Cu/TiO_2_-NC, a procedure was used according to Dulski et al. [49]. Firstly, a water solution of TiO_2_-NPs (20 nm; US Research Nanomaterials Inc., Houston, TX, USA) was prepared at a concentration of 10%. Next, to prepare the surface of TiO_2_-NPs, the alkaline reducer (10% NaOH) and stabilizer (25% ammonia) were used. Then, a water solution of copper acetate was added dropwise into the titanium oxide mixture and the colloidal suspension was formed. Next, in order to reduce the copper ions, ascorbic acid was added until the colour of mixture changed to orange (related to the formation of metallic Cu-NPs on the surface of the TiO_2_ matrix). Finally, Cu/TiO_2_-NC as a nanopowder product was obtained via filtration through a polyethylene filter and drying at room temperature. 

### 4.2. Determining Antibacterial Activity of NMs

Minimum bactericidal concentration (MBC), minimum inhibitory concentration (MIC) and half maximal inhibitory concentration (IC_50_) were determined according to Bagchi et al. [27] and Nowak et al. [50]. For this purpose, the bacteria were cultivated in a liquid Luria−Bertani mix medium (LB mix; tryptone 10 g L^−1^, NaCl 10 g L^−1^, yeast extract 5 g L^−1^) for 3−4 h (37 °C, 130 rpm) in order to reach the mid-exponential growth phase. Next, the bacterial cultures were centrifuged (4 °C, 30 min, 5000× *g*) and the resulting pellets were suspended in sterile 0.85% NaCl. The bacterial suspension was used to inoculate the LB mix medium to achieve OD_600_ = 0.1 (~10^7^ CFU mL^−1^). In the next stage, NMs were added individually to the bacterial cultures in a concentration range of 10 to 1500 mg L^−1^ and were then incubated by shaking (130 rpm) for 24 h at 37 °C. Then, 100 μL of the bacterial suspension from each culture were 10-fold serial diluted in 0.85% NaCl, plated on solid LB and incubated for 24 h at 37 °C. The control samples were bacterial cultures without NMs. The number of bacteria was expressed as the CFU per mL. The concentration corresponding to the MIC and MBC was determined based on the mortality rate using the formula of Bagchi et al. [27], assuming that a 99% growth inhibition of the bacteria corresponded to the MIC value and 100% to the MBC value. The IC_50_ of the tested NMs against *E. coli* and *B. subtilis* was calculated using the Prism 5 tool (GraphPad Software, San Diego, CA, USA). 

### 4.3. Determining the Activity of Catalase, Peroxidase, Superoxide Dismutase and Dehydrogenases

Bacteria from the log phase were transferred to a sterile LB medium supplemented with the appropriate NM (IC_50_) to obtain OD_600_ = 0.1 (~10^7^ CFU mL^−1^) and then incubated at 37 °C for 24 h. Next, the bacterial cultures were centrifuged (4 °C, 5000× *g*), the obtained biomasses were suspended in a 50 mM phosphate buffer and subjected to ultrasonication (20 kHz, six rounds for 15-s pulse and 30-s intervals) at 4 °C using a Vibra Cell (USA) [51]. After centrifugation (4 °C, 20 min, 15,000× *g*), the cell-free extracts were used to measure the activity of CAT, PER and SOD. The CAT activity was measured according to Banerjee et al. [52] and Muniswamy et al. [53] at λ = 240 nm for 3 min as the decrease in the absorbance of H_2_O_2_ (*ɛ* = 36,000 dm^3^ · mol^−1^ · cm^−1^). The PER activity was determined using the enzymatic assay of peroxidase (Sigma-Aldrich) with pyrogallol as the substrate (λ = 420 nm, 3 min). The SOD activity was assayed spectrophotometrically at 450 nm by reducing the tetrazolium salt using a SOD assay kit (cat. 19160, Sigma-Aldrich) and calculated according to Zhang et al. [54]. The protein concentration in the crude extract was calculated according to Bradford [55] with lysozyme as the standard. The specific activity of CAT, PER and SOD was expressed as U · mg^−1^ of protein. The dehydrogenase (DEH) activity was measured using the colorimetric method of Nweke et al. [56] with 2,3,5-triphenyltetrazolium chloride (TTC) as the artificial terminal hydrogen acceptor in the electron transport chain, which was reduced to red-coloured triphenylformazan (TPF) (λ = 485 nm). The activity of DHA was expressed as μg TPF h^−1^ mL^−1^. 

### 4.4. Determining the Effect of NMs on the Bacterial Fatty acid Composition

Fatty acids were isolated directly from the bacterial cells exposed to individual NMs for 24 h according to Sasser [57]. The sample processing included five steps: harvesting (40 mg of bacterial biomass), saponification (5 g sodium hydroxide, 150 mL methanol and 150 mL distilled water), methylation (325 mL 6N hydrochloric acid and 275 mL methyl alcohol), extraction (200 mL hexane and 200 mL methyl tert-butyl ether) and a base wash (0.8 g sodium hydroxide in 900 mL distilled water). All of the obtained fatty acid methyl esters (FAMEs) were analysed using a gas chromatograph (Agilent 7820A, USA), which was equipped with an FID detector and an Ultra 2-HP capillary column (cross-linked 5% phenyl methyl silicone, 25 m, 0.2 mm id and 0.33 μm film thickness), and the TSBA library (ver. 6.2B) (Sherlock Microbial Identification System software, MIDI Inc., Newark, DE, USA). For the detailed analysis, all of the FAMEs were divided into two groups: saturated (straight-chain, branched, hydroxy, cyclopropane) and unsaturated fatty acids.

### 4.5. Imaging of the NMs and Bacterial Cells Using Scanning Electron Microscopy

To evaluate the size and morphology of the bacterial cells, they were visualized before and after treatment using scanning electron microscopy (SEM), in accordance with the methodology of Karcz et al. [58]. The cell surface mapping and interaction of the bacterial membrane, with visualized NMs at the microstructural level, were analysed using energy dispersive X-ray spectrometry (EDS). First, the bacterial cell cultures exposed to NMs (IC_50_) were suspended in 3% glutaraldehyde and incubated at 4 °C for 4 h. Next, the glutaraldehyde was discarded and the fixed cells were washed three times with distilled water. The samples were dehydrated in an increasing ethanol series (30%, 50%, 70%, 80%, 90%, 95% and 100%) by incubating them for 10 min in each alcohol concentration at room temperature. The lyophilisation of the microbial cell was replaced by a drying method using 100% hexamethyldisilazane (HMDS) for 10 min [59]. The dried samples were transferred to graphite tape and coated with technical gold in a Pelco SC-6 sputtering machine (Ted Pella Inc., Redding, CA, USA). The prepared samples were imaged in SEM with field emission (JEOL JSM-7100F, Tokyo, Japan) at an accelerating voltage of 15 kV and a vacuum of 9.6 × 10^−5^ Pa.

### 4.6. Microbial Assay for Risk Assessment (MARA)

A MARA 96-well plate was independently prepared for each type of NM. Aqueous NM solutions were introduced into rows B−G of the wells (except for row A, into which sterile H_2_O was introduced), thus obtaining the final concentrations: 5, 50, 125, 250, 500 and 1000 mg L^−1^. After an 18-h incubation at 30 °C, the plates were scanned with an HP PrecisionScan Pro scanner (Palo Alto, CA, USA) and analysed using MARA software (MARA Protocol WI-NC-237, NCIMB Ltd., Aberdeen, UK). The toxicity of the tested NMs was assessed based on the microbial toxic concentration (MTC), which was calculated as follows [60,61]:

(1) $$MTC=\;c_{min}\cdot d^{\left(p_{tot}-p_{0}\right)-1}$$

where: *c_min_*—the lowest concentration of the toxicant in the gradient, *d*—dilution factor, *p_tot_*—the sum of the pellet sizes in the gradient of the Cu-NPs, TiO_2_-NPs and Cu/TiO_2_-NC, *p*_0_—pellet size in the control.

### 4.7. Statistical Analysis

All experiments were performed in three replicates and the data is presented as the mean ± the standard deviation (SD). The differences in the experimental groups were followed up by using the one-way ANOVA and post hoc Tukey’s honest significant difference test (HSD), in which different letters indicate significant differences using *p* < 0.05 for the threshold of statistical significance. Additionally, principal component analysis (PCA) of the FAME profiles with a relative contribution of >1.5% of the fatty acids with a statistical significance (*p* < 0.05) was used to identify any shifts in the composition of the whole cell-derived fatty acids. For the statistical and graphical analyses of the obtained data, the STATISTICA 13.1 software package (Dell Inc., Austin, TX, USA) and MS Office 2010 (Microsoft Inc., Redmond, WA, USA) was used.

## 5. Conclusions and Future Perspectives

This study demonstrated that the biocidal properties of Cu/TiO_2_–NC are strain dependent, with a greater biocidal activity against reference *E. coli* rather than *B. subtilis*. Moreover, it showed no toxic effects on 10 other taxonomically diverse microbial species. This partially verifies the potential use of Cu/TiO_2_-NC as supplements in future commercial products and possible industrial processes. This research provided also new insight into the biological action of NMs through significant changes in the antioxidant activity, fatty acid composition and specific interactions with bacterial outer layers. To get a deeper insight into the mechanisms of nanostructure action on bacterial cell, our current research is focused on the in vivo gene expression involved in oxidative stress pathways, the level of free radical generation and cellular respiration, as well as molecular-level interactions between NMs and chemical groups on the cell surface.

## Figures and Tables

**Table 1:** The values of MIC, MBC and IC_50_ (mg L^−1^) of the tested NMs against the reference *E. coli* and *B. subtilis* strains.

Type of Nanomaterials	*Escherichia coli* ATCC 25922			*Bacillus subtilis* ATCC 6633		
	MIC	MBC	IC_50_	MIC	MBC	IC_50_
Cu/TiO_2_-NC	500	500	100.61	525	575	464.22
Cu-NPs	575	600	506.35	40	50	4.84
TiO_2_-NPs	500	500	102.16	575	1000	95.83

**Table 2:** The average MTC values (mg L^−1^) for the tested NMs in MARA test.

Type of Nanomaterial	Strain											MTC_av._
	1 (G+)	2 (G-)	3 (G-)	4 (G-)	5 (G+)	6 (G-)	7 (G+)	8 (G+)	9 (G-)	10 (G-)	11	
Cu/TiO_2_-NC	>1000	>1000	>1000	>1000	>1000	>1000	973	>1000	>1000	>1000	>1000	>1000
Cu-NPs	555	>1000	986	683	747	423	463	376	790	97	>1000	559
TiO_2_-NPs	>1000	>1000	>1000	>1000	>1000	>1000	>1000	>1000	>1000	>1000	>1000	>1000

1—*Microbacterium* spp., 2—*Brevundimonas diminuta*, 3—*Citrobacter freundii*, 4—*Comamonas testosterone*, 5—*Enterococcus casseliflavus*, 6—*Delftia acidovorans*, 7—*Kurthia gibsonii*, 8—*Staphylococcus warneri*, 9—*Pseudomonas aurantiaca*, 10—*Serratia rubidaea*, 11—*Pichia anomala*; G+—Gram-positive strain, G—Gram-negative strain.

## Abbreviations

NMs	Nanomaterials
Cu-NPs	Copper nanoparticles
TiO_2_-NPs	Titanium dioxide nanoparticles
Cu/TiO_2_-NC	Copper-titanium dioxide nanocomposite
MBC	Minimum bactericidal concentration
MTC	Microbial toxic concentration
MIC	Minimum inhibitory concentration
IC_50_	Half maximal inhibitory concentration
CAT	Catalase
PER	Peroxidase
SOD	Superoxide dismutase
DEH	Dehydrogenases
FAME	Fatty acid methyl esters
ROS	Reactive oxygen species
